# Design Procedure and Experimental Verification of a Broadband Quad-Stable 2-DOF Vibration Energy Harvester

**DOI:** 10.3390/s19132893

**Published:** 2019-06-29

**Authors:** Abdelhameed A. A. Zayed, Samy F. M. Assal, Kimihiko Nakano, Tsutomu Kaizuka, Ahmed M. R. Fath El-Bab

**Affiliations:** 1 Department of Mechatronics and Robotics Engineering, Egypt- Japan University of Science and Technology (E-JUST), Alexandria 21934, Egypt; 2 Institute of Industrial Science, The University of Tokyo, 4 -6-1 Komaba, Meguro-ku, Tokyo 153-8505, Japan; 3 On leave from Department of Production Engineering and Mechanical Design, Tanta University, Tanta 31511, Egypt; 4 On leave from Department of Mechanical Engineering, Assiut University, Assiut 271516, Egypt

**Keywords:** 2-DOF, multi-stability, nonlinear energy harvesting, piezoelectric, magnetic interaction

## Abstract

Vibration-based energy harvesters brought the idea of self-powered sensors to reality in the past few years. Many strategies to improve the performance of linear vibration energy harvesters that collect energy over a limited bandwidth have been proposed. In this paper, a bi-stable two degrees of freedom (2-DOF) cut-out vibration energy harvester employing a pair of permanent magnets is designed through a proposed design methodology. Based on this methodology, the nonlinear harvesters can be optimally designed such that the bandwidth can be widened for a targeted output voltage. The proper selection of the harvester parameters as well as the gap distances between the tip and the fixed magnets are the bases of this methodology. The mathematical modeling of the proposed harvester and the formula for the potential energy between the tip and the fixed magnets are presented. Additionally, to enhance the performance of the bi-stable energy harvester (BEH), a quad-stable energy harvester (QEH) was configured by adding more fixed magnets. Experiments were performed to validate the numerical simulations and the results showed that, the simulation and experimental results are consistent. The results indicate that, the QEH covers a wider bandwidth than the BEH and based on a figure of merit the QEH shows the best performance among many harvesters presented in the literature.

## 1. Introduction

Energy harvesting from vibrations has become a potential technology to power portable and wireless small electronics instead of batteries. Vibration energy harvesters eliminate the limitations of using batteries in terms of long-term usage and the disposal. A transduction mechanism is required to convert the vibration energy to electricity. The most common transduction mechanisms are the electrostatic, electromagnetic, and piezoelectric mechanisms [1,2,3,4]. Among the aforementioned mechanisms, the piezoelectric material is able to convert the strain energy to electricity directly. Also, it can be easily integrated to the harvesting system especially for wearable devices and considered as flexible electronics [5,6]. Moreover, many research works were oriented toward the rectification, power management, and maximum power tracking so that the generated power can be easily used for wireless sensors [7,8,9]. In its simplest form, the single DOF (SDOF) linear vibration energy harvesting system consists of a cantilever beam that carries a proof mass and has a piezoelectric element attached to its root [10]. These types of harvesters perform optimally when the frequency of the vibration source matches that of the natural frequency of the harvester. Since ambient vibrations are frequency variant, linear SDOF vibration energy harvesters have a limited efficacy and very narrow bandwidth.

To overcome this shortcoming of the linear vibration energy harvesters, vibration energy harvesters with tunable resonance frequency were developed [11,12,13]. The main disadvantages of these harvesters are the complex design and low efficiency because of power consumed for the controller and the delay time of the controller to respond to the changes in the ambient frequency of the active harvesters. Other than those tunable harvesters, array of harvester systems were used to cover a wider bandwidth than that of the linear energy harvesters [14,15]. Each harvester of this array has a different natural frequency, so each one of these harvesters works only at its own natural frequency, which limits the efficiency of this harvester system. Moreover, multimodal vibration energy harvesters that have close resonant modes of vibration were presented to improve the bandwidth of the harvesters [16]. Also different designs to enhance the output of the harvesters such as compliant mechanisms and dual film structured piezoelectric harvesters were presented [17,18].

Additionally, the linear 2-DOF vibration energy harvesters have been used instead of SDOF ones to widen the effective bandwidth of the harvester since they have two resonant peaks [19]. Unfortunately, the response in-between the two peaks is very low, which means that the harvester cannot cover the overall bandwidth between the two peaks. A 2-DOF linear vibration energy harvester was presented in [20] in which two configurations were analytically investigated. One of those configurations has a piezoelectric patch between the first mass and the base, while the other has the piezoelectric patch between the two masses. According to the results of the power response, the first configuration was able to achieve two close effective peaks. After that, the same system was extended to an n-DOF configuration to increase the number of peaks. Another 2-DOF vibration energy harvester that was designed to achieve two close resonant peaks was proposed and experimentally studied in [21]. The structure of this harvester was based on the cut-out structure which is more compact than the conventional 2-DOF structures.

Moreover, the concept of nonlinearity using magnetic interaction was introduced to enhance the performance of the linear vibration energy harvesters in terms of the wide bandwidth and large output power. The Duffing type oscillator, which is the most common nonlinear SDOF harvester, was experimentally investigated under harmonic and colored noise excitations in [22]. In comparison with the linear SDOF energy harvester, the Duffing harvester showed higher output power than that of the linear one. A different study was carried out in [23] based on an analytical approach to investigate the performance of the uni-modal Duffing type scavenger subjected to random forced excitations such as white Gaussian and colored excitations. It was concluded from the results of this work that the uni-modal Duffing type scavenger is not effective for random forced excitations. Also, the performance of a bi-stable Duffing oscillator employing permanent magnets was theoretically and experimentally compared with that of the linear energy harvester in [24]. It was shown that the bi-stable harvester outperforms the conventional one in terms of a larger power over a wide range of frequencies. Another SDOF bi-stable energy scavenger that has asymmetric potential wells was introduced in [25]. This asymmetry was compensated by the influence of gravity on the cantilever beam due to the inclination of the system by a bias angel. The experimental performance of the harvester was effectively enhanced by this method. A tri-stable SDOF harvester that has three potential wells was numerically and experimentally studied in [26]. The restoring force of this harvester was modeled as a high order polynomial in terms of the distance between the fixed and movable magnet. Based on the experimental study, this harvester was able to cover a wider bandwidth than that of the linear harvester. Furthermore, a novel quad-stable SDOF energy harvester with shallower potential wells than that of the bi-stable one for the same separation distance between the magnets was developed in [27]. The experimental results verified that, the tip mass can easily snap through all the potential wells, leading to more power generation and wider bandwidth. Another design based on an arc-shaped cantilever beam to achieve a bi-stable energy harvester was theoretically and experimentally studied in [28]. The results showed that the nonlinear harvester performs better than the linear harvester.

To further improve the performance of nonlinear energy harvesters, nonlinearity was incorporated in 2-DOF systems to widen the operated bandwidth of the harvester and maintain large output power. For instance, a SDOF bi-stable harvester was extended to a 2-DOF system by adding a second linear SDOF oscillator to enlarge the effective bandwidth of the harvester and amplify the output power in [29]. Moreover, a pair of cantilevers, one inside the other, composing a 2-DOF system, was introduced in [30]. Each tip mass of the system carries a permanent magnet to cause magnetic interaction and present nonlinearity in the system. A bi-directional magnetically coupled dual beam bi-stable harvester to scavenge energy from irregular vibrations was reported in [31]. The simulation results which were verified experimentally showed that, the proposed harvester can harvest energy in different directions better than that of the linear system. Additionally, a harvester composed of two inverted cantilever beams coupled magnetically in a 2-DOF system to harvest energy from rotating environments was presented in [32]. The experimental results showed that, the harvester can harvest energy at low rotating speeds over several frequency bands effectively. For the sake of enhancing the bandwidth of the 2-DOF cut-out harvester that was formerly studied in [21], a fixed permanent magnet was introduced to oppose another magnet attached to the mass of the secondary beam in [33]. The performance of this nonlinear 2-DOF configuration was evaluated by carrying out an experimental parametric study. For an optimal configuration, the nonlinear system needs to be able to eliminate the deep valley in-between the two resonant peaks of the linear system and achieve broader bandwidth. Also, the simulation results based on a lumped parameter model of the harvester were compared to the experimental results and the proposed model predicted the trend of broadband respond successfully. Another theoretical model based on a distributed parameter model to predict the performance of the linear and mono-stable behavior of the cut-out 2-DOF energy harvester was developed in [34]. The analytical results obtained based on this theoretical model were compared with the experimental results presented in [33] and a close matching between the results was shown. A new design of a nonlinear multi-degree of freedom was proposed in [35] based on linear element coupling. The numerical investigation of this harvester showed that, the systems composed of two and three harvesters outweigh their linear counterparts in terms of the output power. 

Based on the previous discussions, it is clear that, linear energy harvesters have very limited bandwidth and they are very sensitive to the ambient frequency. Also, the 2-DOF linear energy harvesters suffer from the existence of the deep valley in-between the two resonant peaks. On contrary, the nonlinear energy harvesters have presented potential advantages for improving the performance of device in terms of the wide bandwidth and the amplitude of the generated voltage. Additionally, the nonlinear energy harvesters are more tolerant to the variations in excitation frequency. For the sake of addressing the aforementioned problems, this paper presents a development of a 2-DOF cut-out structure-based nonlinear BEH for further improvement of the generated voltage and the bandwidth of the harvester. Additionally, by adding more magnets, a QEH characterized by lower potential barriers and wide span between the stable points of the system’s potential energy curve is developed. Furthermore, an experimental comparative study between the two nonlinear systems of the 2-DOF cut-out harvester is carried out to show the superiority of the quad-stable system over the bi-stable one in terms of the wide bandwidth. What is more important is that, a systematic approach of designing the nonlinear harvester parameters to target a specific frequency range is presented. This paper is organized as follows. In Section 2, the harvester system description and the dynamic modeling, as well as the calculation of the generated voltage are presented. In Section 3, the steps towards a systematic design methodology of the harvester for a specific frequency range are elaborated. In Section 4, the experimental setup with its details will be presented. Also, the numerical simulation as well as the experimental validation will be carried out. Finally, conclusions are followed in Section 5.

## 2. System Structure and Modeling

### 2.1. Harvester Structure and Description

**Figure 1:**
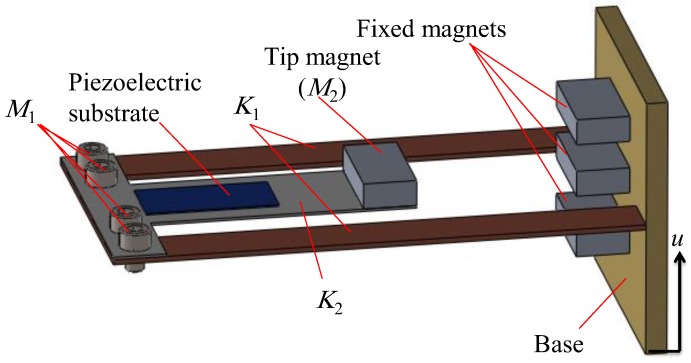
The proposed harvester.

The 2-DOF vibration energy harvester proposed in this paper is shown in Figure 1. The inner beam with length *L*_2_ is enclosed by the outer beam with length *L*_1_ to form the cut-out structure characterize by two close resonant peaks. The first stiffness *K*_1_ and the second stiffness *K*_2_ of the system are represented by the outer and inner beams, respectively. The outer beam carries the first mass *M*_1_ at its free end, while the second mass *M*_2_ that incorporates the tip magnet is attached to the free end of the inner beam. This tip magnet is repelled by three identical permanent magnets fixed to the base. A piezoelectric patch for generating the output voltage is attached to the root of the inner beam. The system is subjected to a harmonic base excitation acceleration of the form *ü =* −*ω^2^ U sin (2πft)* in which *ω = 2πf* where *f* is the frequency of the excitation and *U* is the amplitude of the harmonic excitation *u*.

### 2.2. Theoretical Modeling

The dynamic equations of the cut-out 2-DOF system shown in Figure 1 in a matrix form of the damped forced system excluding the effect of the piezoelectric patch can be written as follows:

(1) $$M\ddot{y}+C\dot{y}+Ky=F.$$

where $M=\left[\begin{array}{cc} M_{1} & 0\\ 0 & M_{2} \end{array}\right]$ is the mass matrix, $K=\left[\begin{array}{cc} K_{11} & K_{12}\\ K_{21} & K_{22} \end{array}\right]$ is the stiffness matrix, $C=\left[\begin{array}{cc} C_{11} & C_{12}\\ C_{21} & C_{22} \end{array}\right]$ is the damping matrix which is assumed to be proportional to the mass matrix *M* and the stiffness matrix *K* by the relation C=τ(M+K) in which *τ* is the proportional constant, $F={\left[\begin{array}{cc} -M_{1}\ddot{u} & -M_{2}\ddot{u} \end{array}\right]}^{T}$ is the force vector of the system and $y={\left[\begin{array}{cc} y_{1} & y_{2} \end{array}\right]}^{T}$ is the generalized coordinates vector in which *y*_1_ and *y*_2_ are the displacements of *M*_1_ and *M*_2_ with respect to the equilibrium position, respectively. 

The relation between the two natural frequencies *ω*_1_ and *ω*_2_ and the system parameters *K*_1_, *K*_2_, *M*_1_ and *M*_2_ can be obtained from the characteristic equation of the system. The two natural frequencies of the system *ω*_1_ and *ω*_2_ can be derived as follows:

(2) $$\omega_{1}={\left[\frac{\frac{K_{11}M_{2}+K_{22}M_{1}}{M_{1}M_{2}}-\sqrt{{\left(\frac{K_{11}M_{2}+K_{22}M_{1}}{M_{1}M_{2}}\right)}^{2}-4\left(\frac{K_{11}K_{22}-K_{12}K_{21}}{M_{1}M_{2}}\right)}}{2}\right]}^{1/2}$$

(3) $$\omega_{2}={\left[\frac{\frac{K_{11}M_{2}+K_{22}M_{1}}{M_{1}M_{2}}+\sqrt{{\left(\frac{K_{11}M_{2}+K_{22}M_{1}}{M_{1}M_{2}}\right)}^{2}-4\left(\frac{K_{11}K_{22}-K_{12}K_{21}}{M_{1}M_{2}}\right)}}{2}\right]}^{1/2}$$

**Figure 2:**
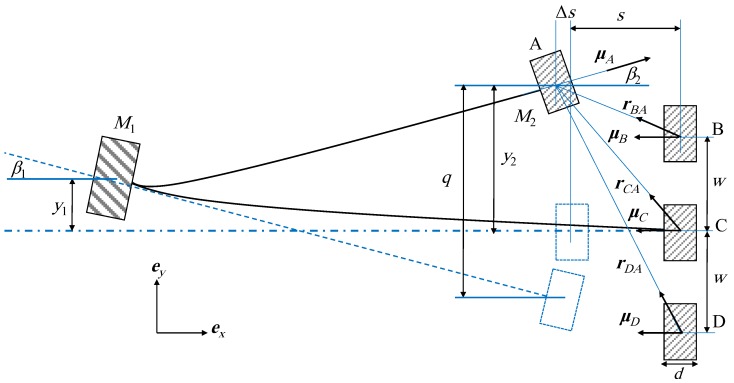
Magnetic moment vectors and displacements of the masses of the harvester cut-out harvester.

By considering the piezoelectric patch to be bonded to the root of the inner beam, the dynamic equations of the system are modified as follows:

(4) $$M_{1}\ddot{y_{1}}=-K_{11}y_{1}-K_{12}y_{2}-C_{11}{\dot{y}}_{1}-C_{12}{\dot{y}}_{2}-\lambda V-M_{1}\ddot{u}$$

(5) $$M_{2}\ddot{y_{2}}=-K_{22}y_{2}-K_{21}y_{1}-C_{22}{\dot{y}}_{2}-C_{21}{\dot{y}}_{1}+\lambda V-M_{2}\ddot{u}$$

(6) $$\frac{V}{R}+C_{p}\dot{V}-\lambda \dot{q}=0$$

where λ is the electromechanical coupling coefficient; *C_p_* represents the capacitance of the piezoelectric patch; *V* is the generated voltage across a resistive load *R* and *q* is the displacement of the second mass *M*_2_ from its free (un-deformed) position as shown in Figure 2. The stiffness matrix for the cut-out structure can be given as follows [33]:

(7) $$K=\frac{\left(6EI_{1}\right)}{\left(4EI_{1}L_{1}^{3}L_{2}^{3}+3EI_{2}L_{1}^{4}L_{2}^{4}\right)}\left[\begin{array}{cc} 2EI_{1}L_{2}^{3}+2EI_{2}L_{1}^{3}+6EI_{2}L_{1}L_{2}^{2}-6EI_{2}L_{1}^{2}L_{2} & -2EI_{2}L_{1}^{3}+3EI_{2}L_{1}^{2}L_{2}\\ -2EI_{2}L_{1}^{3}+3EI_{2}L_{1}^{2}L_{2} & 2EI_{2}L_{1}^{3} \end{array}\right]$$

where *E* is the modulus of elasticity of the material of the beams and $I_{i}=b_{i}\cdot h_{i}^{3}/12$ for *i* = 1, 2 is the moment of inertia for the outer and the inner beams, respectively, in which *b* and *h* are the width and the thickness of the beam, respectively. The same procedure presented in [33] is followed to calculate *q* which yields:

(8) $$q=y_{2}-y_{1}+L_{2}\beta_{1}$$

where *β*_1_ is the angle of rotation of *M*_1_ with respect to the horizontal reference as shown in Figure 2. It can be calculated as follows:

(9) $$\beta_{1}=\gamma_{11}\left(K_{11}y_{1}+K_{21}y_{2}\right)+\gamma_{21}\left(K_{12}y_{1}+K_{22}y_{2}\right)$$

where *γ_ij_* is the angle of rotation of the beam at mass position *j* when a unit force is applied at mass position *i* [36]. Similarly, *β*_2_ shown in Figure 2 can be calculated as follows:

(10) $$\beta_{2}=\gamma_{12}\left(K_{11}y_{1}+K_{21}y_{2}\right)+\gamma_{22}\left(K_{12}y_{1}+K_{22}y_{2}\right)$$

Then *γ* matrix can be derived as follows:

(11) $$\gamma =\left[\begin{array}{cc} L_{1}^{2}/2EI_{1} & (L_{1}^{2}-2L_{1}L_{2})/2EI_{1}\\ (L_{1}^{2}-2L_{1}L_{2})/2EI_{1} & (I_{1}L_{2}^{2}-I_{2}{\left(L_{1}-L_{2}\right)}^{2})/2EI_{1}I_{2} \end{array}\right]$$

Therefore, *q* can be derived in terms of *γ_ij_* as follows:

(12) $$q=\sigma y_{2}-\rho y_{1}$$

where

(13) $$\sigma =1+\left(\gamma_{11}K_{21}+\gamma_{21}K_{22}\right)$$

(14) $$\rho =1-\left(\gamma_{11}K_{11}+\gamma_{21}K_{12}\right)$$

Finally, the displacement *q* and its time derivative can be represented in terms of the generalized coordinates and their time derivatives to be substituted in the system dynamic model.

With the fixed permanent magnets to be added to the system, the nonlinearity is presented in the system and this effect should be incorporated in the governing equations of the system. The effect of adding permanent magnets to the system is represented as added magnetic potential energy to the elastic potential energies of the beams. The magnetic potential energy can be derived as follows.

For the interaction between the tip and the fixed magnets, the permanent magnets are modeled as magnetic dipoles, and the generated magnetic fields by the external magnets B, C, and D on the tip magnet A are calculated as follows [37]:

(15) $$p_{nA}=-\frac{\mu_{0}}{4\pi }\nabla \frac{\mu_{n}\cdot r_{nA}}{{\left|r_{nA}\right|}^{3}}$$

where *n* equals A, B or C, ∇ is the vector gradient operator with respect to *y_2_* and *∆s* which is the horizontal displacement of the tip magnet from its initial state and $\Delta s=\frac{d}{2}\left(1-cos\beta_{2}\right)$ in which *d* is the width of the magnet; *μ_0_* is the permeability constant of space; ***r****_nA_* is the position vector directed from the center of the magnet *n* to the center of the tip magnet A and ***μ****_n_* is the magnetic dipole moment vector of the magnet *n* that is directed perpendicular to the parallel surfaces of the tip magnet and the fixed magnet at their initial state from the center of the fixed magnet to the tip magnet, as shown in Figure 2. From the geometrical configuration, the position vectors are expressed as:

(16) $$r_{BA}=\left(y_{2}-w\right)e_{y}-\left(s+\Delta s\right)e_{x}$$

(17) $$r_{CA}=\left(y_{2}\right)e_{y}-\left(s+\Delta s\right)e_{x}$$

(18) $$r_{DA}=\left(y_{2}+w\right)e_{y}-\left(s+\Delta s\right)e_{x}$$

where *s* is the horizontal separation distance between the initial position of the tip magnet and the initial positions of the fixed magnets and ***e****_x_* and ***e****_y_* are the fixed unit vectors parallel to the upper surface of the two beams in their initial positions and the one perpendicular to it, respectively, as shown in Figure 2.

Also, the magnetic moment vectors of the four magnets can be calculated as follows:

(19) $$\mu_{A}=\left(M_{A}V_{A}\cos\beta_{2}\right)e_{x}+\left(M_{A}V_{A}\sin\beta_{2}\right)e_{y}$$

(20) $$\mu_{n}=-\left(M_{n}V_{n}\right)e_{x}$$

where ***μ****_A_* is the magnetic dipole moment vector of the magnet A; *V_n_* is the volume of the magnet *n* and *M_n_* is the magnetization of the magnet *n* in which the magnetization *M* is considered as an expression of the intensity of the permanent magnetic moment in the ferromagnetic material and has a relation with the surface flux density *B_r_* expressed as:

(21) $$B_{r}=M\mu_{0}$$

The total potential energy of the magnetic field for the quad-stable system due to the effect of the three magnets B, C, and D on the tip magnet A can be derived as follows:

(22) $$Q_{m}\left(y_{2}\right)=-\mu_{A}\cdot p_{BA}-\mu_{A}\cdot p_{CA}-\mu_{A}\cdot p_{DA}$$

By substituting (15)–(20) into (22), the final form of the magnetic potential energy can be given as follows:

(23) $$\begin{array}{c} Q_{m}=\frac{\mu_{0}M_{A}V_{A}}{4\pi }[[\left(\frac{3M_{B}V_{B}(s+\Delta s)[(s+\Delta s)\cos\beta_{2}-(y_{2}-w)\sin\beta_{2}]}{|r_{BA}{|}^{5}}\right)-(\frac{M_{B}V_{B}\cos\beta_{2}}{|r_{BA}{|}^{3}})]\\ +[(\frac{3M_{C}V_{C}(s+\Delta s)[(s+\Delta s)\cos\beta_{2}-(y_{2})\sin\beta_{2}]}{|r_{CA}{|}^{5}})-(\frac{M_{C}V_{C}\cos\beta_{2}}{|r_{CA}{|}^{3}})]\\ +[(\frac{3M_{D}V_{D}(s+\Delta s)[(s+\Delta s)\cos\beta_{2}-(y_{2}+w)\sin\beta_{2}]}{|r_{DA}{|}^{5}})-(\frac{M_{D}V_{D}\cos\beta_{2}}{|r_{DA}{|}^{3}})]] \end{array}$$

where *w* is the separation distance between each two fixed magnets. From (23), the magnitude of the magnetic force *f_m_* between the fixed magnets and the tip magnet in the vertical direction can be written as follows:

(24) $$f_{m}=-\frac{\partial Q_{m}}{\partial \left(y_{2}\right)}$$

The total potential energy of the system which consists of the magnetic potential energy and the potential energy of the two elastic beams is given as follows:

(25) $$Q_{t}=Q_{m}+\frac{1}{2}K_{11}y_{1}^{2}+\frac{1}{2}K_{22}y_{2}^{2}$$

Therefore, the modified governing equations of the system are given as follows:

(26) $$M_{1}\ddot{y_{1}}=-K_{11}y_{1}-K_{12}y_{2}-C_{11}{\dot{y}}_{1}-C_{12}{\dot{y}}_{2}-\lambda V-M_{1}\ddot{u}$$

(27) $$M_{2}\ddot{y_{2}}=-K_{22}y_{2}-K_{21}y_{1}-C_{22}{\dot{y}}_{2}-C_{21}{\dot{y}}_{1}+\lambda V-\frac{\partial Q_{m}\left(y_{2}\right)}{\partial y_{2}}-M_{2}\ddot{u}$$

(28) $$\frac{V}{R}+C_{p}\dot{V}-\lambda \dot{q}=0$$

## 3. Design Methodology of The Harvester

Since the majority of ambient vibrations are distributed over a wide frequency range; so, the main function of a vibration energy harvester is to generate adequate voltage over a wideband of frequencies. In this section a systematic approach for designing the 2-DOF nonlinear harvester parameters to target a specific frequency range and to obtain high generated voltage is presented. The parameters to be obtained by this approach are harvester’s parameters as well as the distances between the magnets to obtain high voltage generation over a wide band of frequencies. 

### 3.1. Harvester Parameters Selection

For the sake of developing a systematic approach for choosing the system parameters for nonlinear systems, it is imperative to discuss the following. For an ambient source of vibration with a certain range of frequencies starting from *f*_a_ and ending at *f*_b_, it is easy to design a linear 2-DOF vibration energy harvester to target this frequency range. This can be achieved by adjusting the parameters of the system; namely, *M*_1_, *M*_2_, *K*_1_ and *K*_2_ to have two natural frequencies for the system that match the frequency range of the ambient vibration. On the contrary, for the nonlinear harvester, the approach is not that simple. However, by careful inspection of the behavior of nonlinear systems, it was shown for the SDOF system that, due to the effect of the magnetic interaction on the system, the stiffness of the system is changed. Consequently, the natural frequency is altered to the left for repulsive magnetic forces while it is altered to the right for attractive magnetic forces. This alteration depends on the gap distance between the tip and the fixed magnets of the system since it has a great influence on the magnetic force. For instance, in [38], the resonance of the linear system was at 26 Hz, while it was altered to 13 Hz for a repulsive magnetic force at a distance of 9.5 mm between the end and the fixed magnet. Also, the peak was shifted to 40 Hz for a gap distance of 7.5 mm between two attractive magnets. Moreover, the resonant peak of the linear harvester was observed at 13 Hz, while the responses of the bi-stable and the tri-stable system were before 12 Hz by using repulsive magnets in [39] and the response at 13 Hz was very low. Additionally, careful observation of the 2-DOF systems which have two resonant peaks, the alteration of the peaks depends on the change of the stiffness matrix of the system due to the magnetic interaction. Also, this alteration is caused mainly to the second resonance frequency when the second beam is mostly affected by the magnetic force. In other words, the second resonant peak is altered while the first peak is almost kept unchanged when the magnetic interaction is applied mainly to the second mass [33,40].

**Figure 3:**
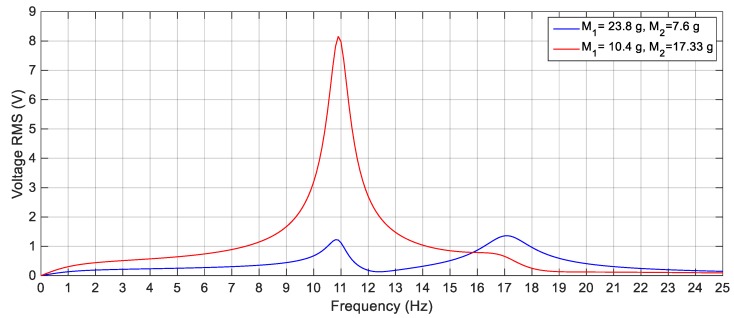
Simulation response of the linear harvester for the two sets of solution of masses at 3 m/s^2^.

For the 2-DOF cut-out structure-based harvester proposed in this paper, the parameters in the linear configuration will selected such that the first natural frequency of the system is chosen the same as the high frequency of the source, *f_b_*. The reason for choosing the first natural frequency of the system the same as the high frequency of the ambient source, *f_b_*, is the alteration of the response of the nonlinear system to the left before the first natural frequency due to the effect of the magnetic interaction between the tip and the fixed magnets that changes the stiffness of the system. Consequently, the nonlinear harvester will be able to scavenge energy over a wideband of frequencies of the source. Moreover, the second natural frequency is chosen in the way that, the two resonant peaks of the linear harvester have high amplitudes. Namely, the two natural frequencies of the harvester in the linear configuration are chosen to avoid the cases with low amplitudes or those with a deep valley in-between the resonant peaks. For instance, referring to (7), it can be noticed that, the stiffness matrix becomes a diagonal matrix with *K*_12_ and *K*_21_ equal zero when *L*_2_ = 2*L*_1_/3. As a result, the 2-DOF system will be decoupled into two SDOF systems. Therefore, this case should be avoided while selecting the harvester parameters in the linear configuration. Generally, since the natural frequencies *ω*_1_ and *ω*_2_ of the 2-DOF system are highly affected by the dimensions and parameters of the system, a careful selection of the masses and dimensions of the beam should be considered to have a good response. The length, width, and thickness of the two beams are selected to calculate the stiffness matrix based on (7) avoiding the ratio of *L*_2_/*L*_1_ = 2/3. After calculating the stiffness matrix and based on a given certain natural frequencies, the masses of the system are calculated using (2) and (3). Solving (2) and (3) together results in two sets of solution for the masses values. One of them makes *ω_1_* higher than *ω_2_* while the other one makes *ω_2_* higher than *ω_1_*. For instance, and for more clarification, assuming the ambient vibration source having a frequency range from 6 Hz to 11 Hz, the natural frequencies of the proposed 2-DOF cut-out harvester are considered to be 14 Hz ± 22% from which the natural frequencies are calculated as 10.9 Hz and 17 Hz. Table 1 shows the geometric and material properties of the used beams, piezoelectric patches and magnets. Based on (7), the stiffness matrix is calculated as $\left[\begin{array}{cc} 117.43 & -16.568\\ -16.568 & 85.598 \end{array}\right]$ N/m. Also, the masses *M*_1_ and *M*_2_ of the system are calculated from (2) and (3) as 23.8 g and 7.6 g for the first set and 10.41 g and 17.33 g for the second set. As mentioned before, the damping matrix is proportional to the mass and the stiffness matrices, with unknown proportional constant, *τ*. Here, for the sake of fair comparison between the simulation and experimental results, the proportional constant, *τ*, is calculated such that, the response of the simulated linear system under excitation amplitude of 3 m/s^2^ is as close as that of the experimental one for the same excitation amplitude. Based on that, the calculated damping matrix is given as $\left[\begin{array}{cc} 0.1292 & -0.0182\\ -0.0182 & 0.0942 \end{array}\right]$ N·s/m. It is worthy to mention that, after applying a magnetic force on the second mass, this magnetic force mainly affects the stiffness of the inner beam. Thus, the first natural frequency is slightly changed, while the second one is altered to the left. Since high performance harvester is characterized by no existence of a deep valley in-between the two resonant peaks, the set of solution for the system masses that makes *ω_2_* higher than *ω_1_* should be chosen. Figure 3 shows the linear response of the 2-DOF cut-out harvester for the two sets of masses. It is clear that, the response for the first set of solution has adequate resonant peaks, while the response for the second set has a very high first peak and relatively low second peak.

### 3.2. Selection of Gap Distances Between Magnets

The dynamic response and the generated voltage of the 2-DOF harvester depend on the potential well depth and the span between the potential wells. So, the potential curves of the harvester are of great importance to study the dynamic performance of such harvesters. The potential well depth is directly related to the gap between the tip and the fixed magnets as well as the distance between the fixed magnets themselves. The effect of these distances on the potential energy of two cases; namely, bi-stable and quad-stable, will be discussed here.

**Figure 4:**
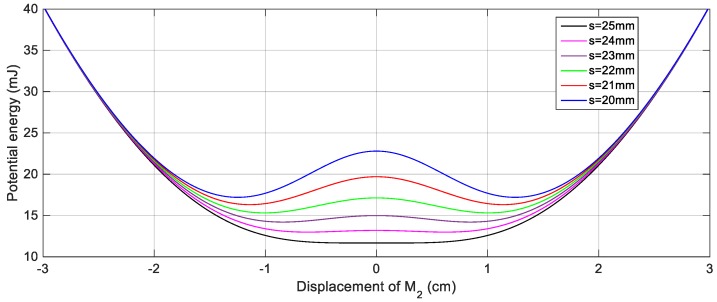
Potential energy curves of the BEH for different gap values between the tip and the fixed magnets.

**Figure 5:**
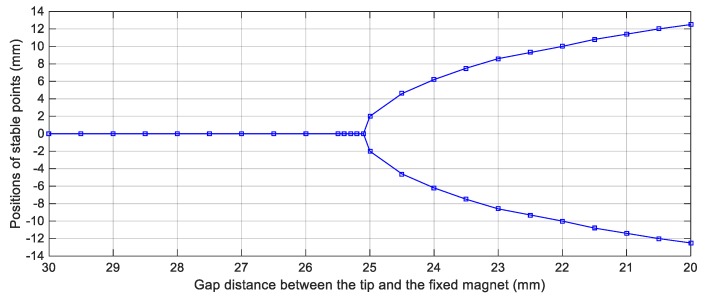
Positions of the stable points against the gap distance values between the tip and the fixed magnets for the BEH.

For achieving the bi-stable configuration, one magnet is placed in front of the tip magnet to introduce a repulsive magnetic force as shown in Figure 1. In order to estimate the suitable gap distance between the tip and the fixed magnets that should be set to achieve the bi-stable configuration, the potential energy curve of this configuration should be examined. Figure 4 shows the simulation results of the potential energy curves of this nonlinear configuration for various gap distances, *s*, between the tip, and the fixed magnets. It is clear from Figure 4 that, decreasing *s* from *s* = 30 mm, the bi-stable configuration is first achieved at *s* = 25 mm. It is also obvious that, the depth of the potential wells is adversely proportional to the distance between the tip and the fixed magnets. In other words, by decreasing the gap, *s*, the potential barrier height increases and as a result, the second mass is confined in one of the potential wells. Moreover, the distance between the two stable points is increased with decreasing the distance, *s*, as depicted in Figure 5 which illustrates the variation of the distance between the two stable points against different values of the distance, *s*. The distance between the two equilibrium positions, the upper and lower positions, is 4 mm for *s* = 25 mm, while this distance reaches 25 mm for *s* = 20 mm as illustrated from Figure 5. Because of this tradeoff between the depth of the potential wells and the distance between the equilibrium positions, there should be a specific distance, *s*, between the tip and the fixed magnets at which the widest bandwidth occurs for a desired output voltage.

**Figure 6:**
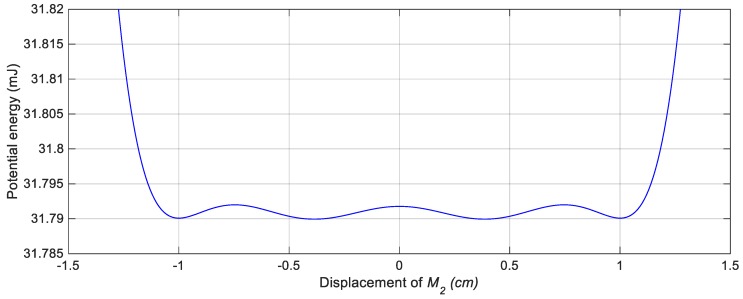
Potential energy curve of the QEH for *s* = 22.8 mm and *w* = 10.84 mm.

In order to configure a quad-stable harvester with its four equilibrium positions, three magnets are fixed in front of the tip magnet as shown in Figure 1. Now the potential energy of this harvester is not only affected by the gap between the tip and the fixed magnets but also, the distance between the centers of the fixed magnets. In order to improve the response of the QEH over the one of the BEH and cover a wider bandwidth, the potential barriers of the QEH should be shallower than that of the BEH. Consequently, the second mass can easily snap through the four potential wells without the need of higher excitations. The potential energy curve of the QEH is shown in Figure 6 for *s* = 22.8 mm and *w* = 10.84 mm which shows that, the potential barriers are lower than that of the bi-stable one. 

From the aforementioned analysis of the potential energy, it can be concluded that, the height of the potential energy barriers and the span between the potential wells are very sensitive to the magnetic gaps *s* and *w*. So, these gaps should be selected carefully to obtain a potential energy curve that can permit for the second mass to travel among all potential wells leading to more voltage generation over a wide bandwidth. 

## 4. Simulation and Experimental Results

### 4.1. Experimental Setup Description

**Figure 7:**
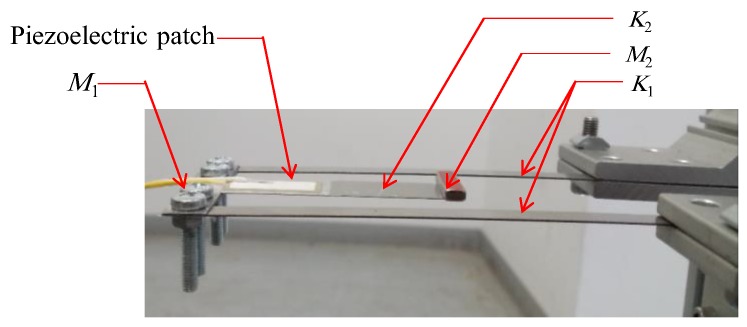
2-DOF cut-out harvester.

**Figure 8:**
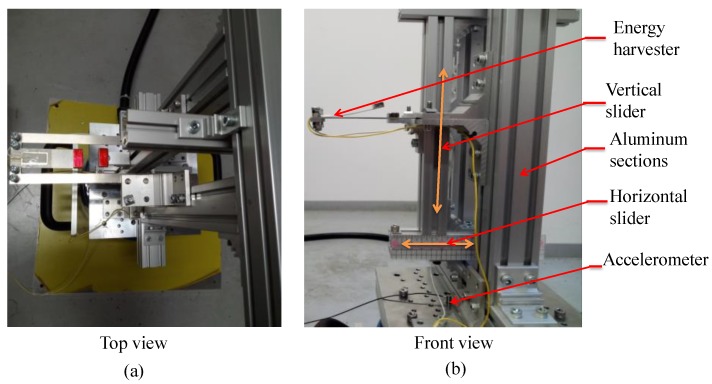
The harvester with the holding frame showing the sliders (**a**) Top view (**b**) Front view.

**Figure 9:**
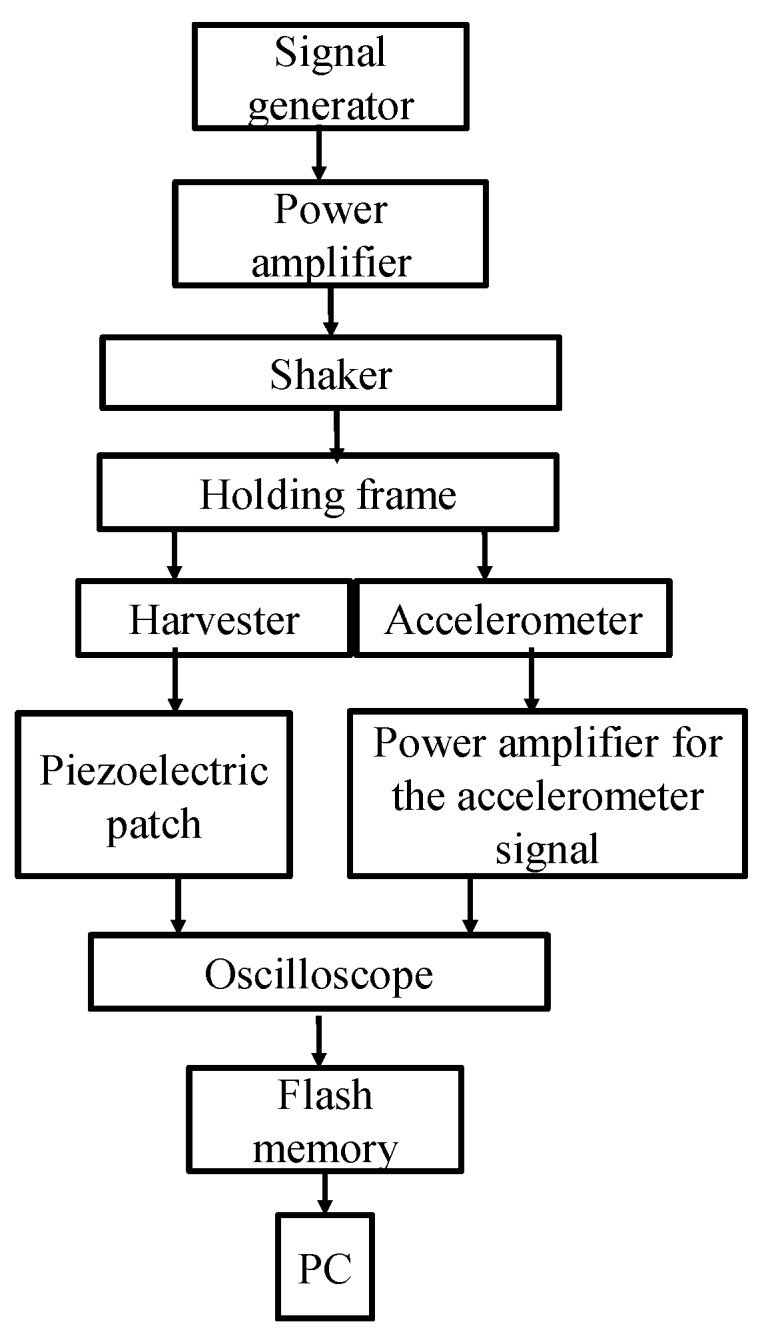
Schematic diagram of the experimental setup.

**Figure 10:**
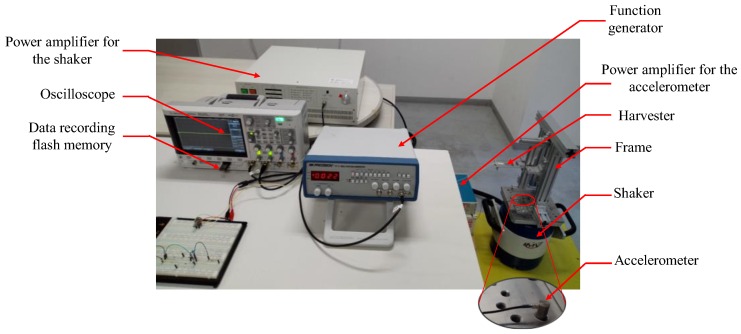
Experimental setup.

An experimental setup is designed and fabricated to investigate experimentally the performance of the proposed harvester in its different configurations of bi-stable and quad-stable, as well as validate the simulation results that are obtained based on the mathematical model of the device. This setup is also developed to show the enhancement of the performance of the harvester in its quad-stable configuration. The constructed 2-DOF harvester is composed of two identical Stainless Steel beams representing the first stiffness and one Stainless Steel beam with different length representing the second stiffness which is located inside the first stiffness as illustrated in Figure 7. Each beam is manufactured separately and then assembled with each other in a cut-out configuration by the means of bolts which are considered as a part of the first mass. The first (outer) beam carries the first mass *M*_1_, while the piezoelectric patch (K2512U1, KINEZ) is attached to the surface of the second (inner) beam which carries the second mass *M*_2_ at its end as shown in Figure 7. This second mass *M*_2_ incorporates the tip magnet which is responsible for introducing the nonlinearity. This harvester is held horizontally by a vertical frame that is fabricated from Aluminum sections (MISUMI Group Inc., Japan). A part of this frame that carries the fixed magnet slides in the horizontal direction to adjust the distance between the centers of the tip and the fixed magnets. The frame has also another part that slides in the vertical direction to adjust the distance between the centers of the fixed magnets that are located vertically in case of quad-stable configuration as depicted in Figure 8. The frame and the harvester are installed on the table of a shaker (m060, IMV CORPORATION, Osaka, Japan) which is considered as the source of the vibrating motion. A function generator (4011A, BK PRECISION, Yorba Linda, CA, USA) is used to generate the exciting signal with a specific frequency. This signal is fed to a power amplifier (MA1, IMV CORPORATION, Osaka, Japan) to amplify the amplitude of the signal transmitted to the shaker. An accelerometer (PV-08A, RION CO., LTD, Tokyo, Japan) that was fixed to the base of the holding frame was used to measure the acceleration of the shaker table on which the frame holding the energy harvester was installed. The output signal of the accelerometer was sent to another power amplifier (RION CO., LTD, Tokyo, Japan) which in turn transfers it to a digital oscilloscope (MSO-X 3014A, Agilent Technologies, Santa Rosa, CA, USA). Once the harvester is excited, the voltage generated by the piezoelectric patch attached to the inner beam is measured by the oscilloscope. The output voltage and the measured acceleration of the accelerometer are saved to a flash memory attached to the oscilloscope for recording the collected data for further processing. The schematic diagram of the experimental setup is shown in Figure 9, while the detailed real experimental setup is shown in Figure 10.

**Figure 11:**
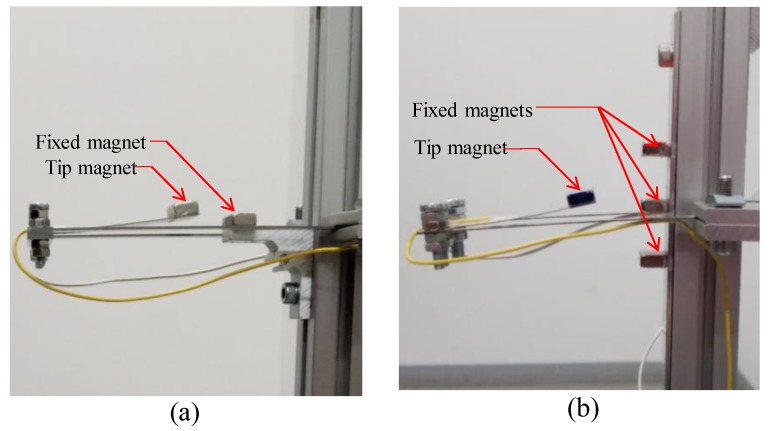
Nonlinear configurations (**a**) bi-stable configuration (**b**) quad-stable configuration.

For the nonlinear systems, the second mass incorporates a permanent magnet to cause magnetic interaction with the other magnets fixed to the frame holding the harvester. A set of NdFeB (NS0126, Magfine, Miyagi, Japan) permanent magnets is used to achieve the BEH and the QEH configurations. Figure 11a shows the configuration of the bi-stable harvester which is achieved by one fixed magnet that opposed the tip magnet, while the quad-stable configuration is achieved by adding three fixed magnets facing the tip magnet as shown in Figure 11b. 

### 4.2. Experimental Procedure

In order to validate the proposed methodology experimentally, experiments are carried out to study the performance of the proposed 2-DOF cut-out harvester for linear, bi-stable, and quad-stable configurations designed based on the proposed methodology. The harvester is subjected to various harmonic excitation levels having amplitude ranges from 2.5 m/s^2^ to 4 m/s^2^. The frequency of the excitation is increased gradually from 3 Hz to 20 Hz. The time series of the output voltage was recorded for each frequency and the RMS of this signal was calculated. The calculated RMS value of the voltage was plotted against the corresponding frequency to get the response curves of the harvester. Also, the effect of varying the separating distance between the tip and the fixed magnets on the response of the BEH and the QEH was studied. After that, a comparison was carried out between the BEH and the QEH in terms of the output voltage and the effective bandwidth for the same gap distance between the tip and the fixed magnets to show the superiority of the performance of the QEH over the one of the BEH.

### 4.3. Results and Discussion

#### 4.3.1. Linear Energy Harvester

**Figure 12:**
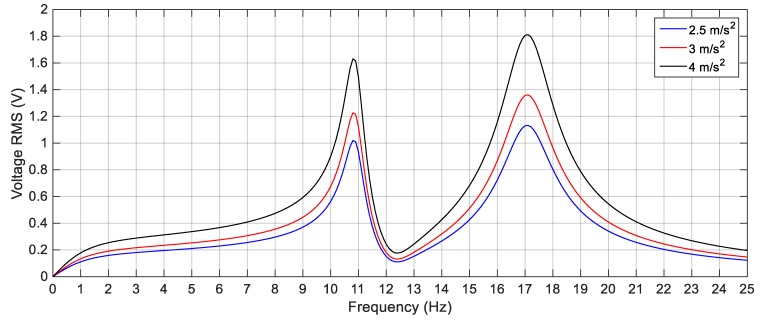
Simulation responses of the LEH for different excitation amplitudes.

**Figure 13:**
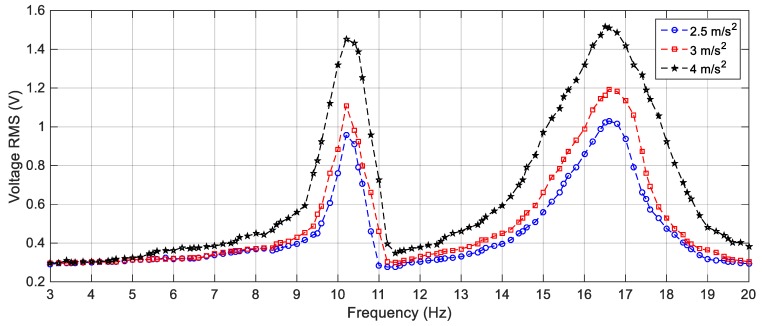
Experimental responses of the LEH for different excitation amplitudes.

**Figure 14:**
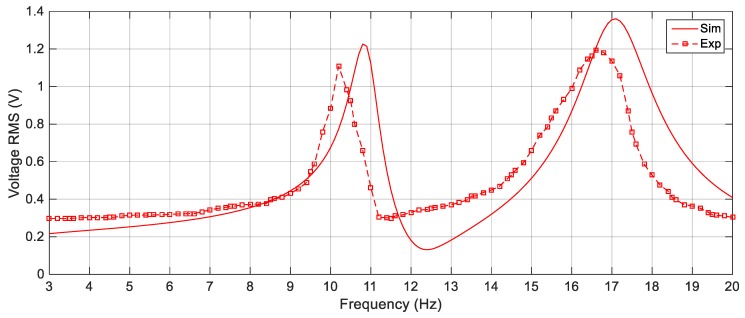
Simulation vs. Experimental responses of the LEH for 3 m/s^2^.

First, the linear harvester is simulated with the parameters given in Table 1 and the first set of masses which are 23.8 g and 7.6 g for *M*_1_ and *M*_2_, respectively. The response of the proposed simulated cut-out linear harvester is shown in Figure 12 for different excitation values of 2.5 m/s^2^, 3 m/s^2^ and 4 m/s^2^, while the experimental ones for the same excitation amplitudes is illustrated in Figure 13. It is clear from Figure 13 that, there is a deep valley in-between the two resonant peaks at which the generated voltage is very low. By increasing the amplitude of the excitation, the experimental output voltage increases, but the deep valley between 11 Hz and 12 Hz still exists. The simulation versus the experimental results for the LEH subjected to 3 m/s^2^ are presented in Figure 14 from which it is obvious that, the experimental and simulation results are consistent. Also, the natural frequencies of the experimental results are slightly shifted from their simulated counterparts; namely, the simulated natural frequencies are calculated numerically as 10.8 Hz and 17.1 Hz, while the experimental natural frequencies are recorded at 10.2 Hz and 16.8 Hz for the first and the second natural frequencies, respectively. This shift in the natural frequencies was due to the manufacturing defects; namely, the errors in measuring the dimensions of beams and masses which cause errors in calculating the damping matrix which is based on the mass and stiffness matrices.

#### 4.3.2. Bi-stable Energy Harvester

**Figure 15:**
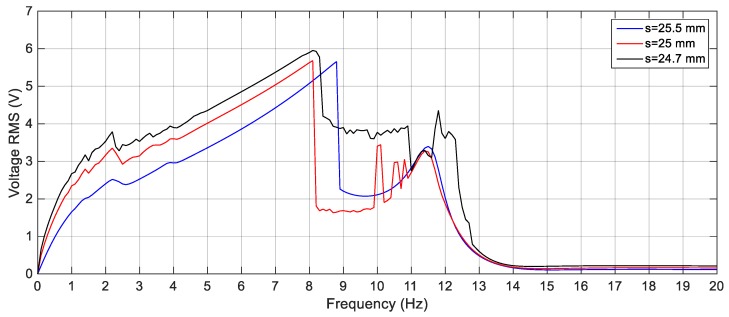
Simulation responses of the nonlinear harveter for different gap distances, *s* = 25.5, 25 and 24.7 mm.

To study the performance of the BEH, one magnet is placed in front of the tip magnet to introduce repulsive magnetic force as shown in Figure 11a. The behavior of the BEH is studied under different excitation levels and with different gaps between the tip and the fixed magnets which is decreased gradually till the moment at which the second mass iswas confined in one of the potential wells. The simulations are carried out to obtain the response of the BEH for different values of *s* that ranges from 25.5 mm to 22 mm. Figure 15 shows the simulated voltage responses of the harvester for different gaps between the tip and the fixed magnets as 25.5, 25, and 24.7 mm under excitation amplitude of 3 m/s^2^. At gap *s* of 25.5, mm the configuration is not bi-stable; namely, the response increases gradually from 0 Hz to 8.8 Hz then drops suddenly and then has another peak of 11.6 Hz. By decreasing the gap to 25 mm, the bi-stable configuration is achieved as indicated in Figure 15. Although the response increased more till 8.1 Hz, the voltage becomes lower than that of 25.5 mm gap and voltage fluctuations start to appear between 8.1 Hz and 11.5 Hz. This is due to the calculation of RMS of the output voltages that are not harmonic. 

**Figure 16:**
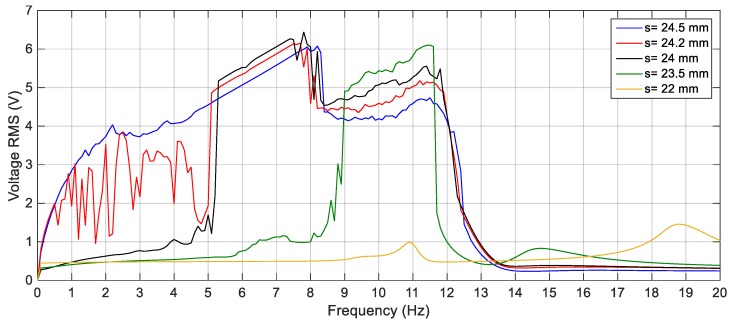
Simulation responses of the BEH for different gap distances, *s* = 24.5, 24.2, 24, 23.5 and 22 mm.

**Figure 17:**
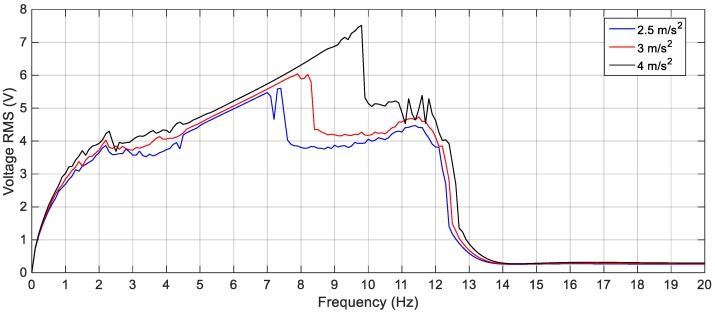
Simulation responses of the BEH for different excitation amplitudes (2.5 m/s^2^, 3 m/s^2^ and 4 m/s^2^), *s* = 24.5 mm.

**Figure 18:**
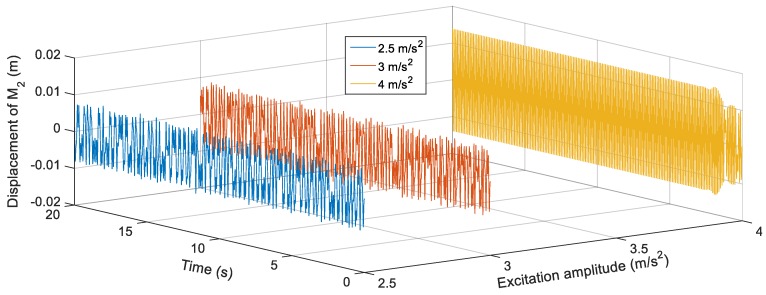
Simulated displacements of the second mass *M*_2_ for different excitation amplitudes (2.5 m/s^2^, 3 m/s^2^ and 4 m/s^2^) at 9 Hz and *s* = 24.5 mm.

**Figure 19:**
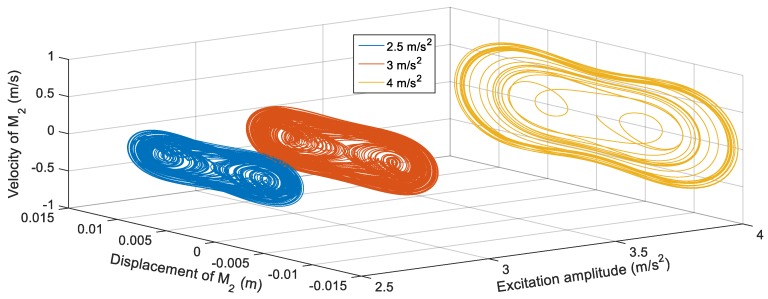
Phase portraits of the second mass *M*_2_ for different excitation amplitudes (2.5 m/s^2^, 3 m/s^2^ and 4 m/s^2^) at 8 Hz and *s* = 24.5 mm.

**Figure 20:**
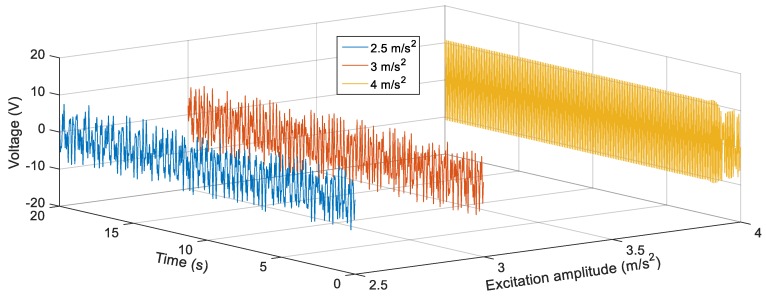
Simulated output voltages for different excitation amplitudes (2.5 m/s^2^, 3 m/s^2^ and 4 m/s^2^) at 9 Hz and *s* = 24.5 mm.

Figure 16 shows the voltage response of the BEH for other values of *s*, where the voltage fluctuations start to appear because the second mass can snap through between the two potential wells at some frequencies and confined in one of the potential wells at other frequencies. For the sake of obtaining the proper gap distance, *s* that achieves the widest bandwidth for a targeted output voltage, a targeted output voltage of 4 V is considered. The BEH with a gap distance of 24.5 mm is able to achieve a bandwidth of 8.3 Hz, while the bandwidth is 6.8 Hz for *s* = 24 mm. It is obvious that, by a further decrease of *s*, a narrow bandwidth is achieved at 4 V, while higher voltages were obtained. When the fixed and the tip magnets are as close as *s* = 22 mm, the second mass is confined in one of the potential wells for all frequencies and the response had a narrow bandwidth. In Figure 16, small peaks between 14 Hz and 20 Hz for different values of the gap distance can be noticed. These peaks are due to the increase of the displacement of the second mass at these higher frequencies, while it is restricted in one potential well without snapping through the other potential well. From the simulation results, it is clear that the widest bandwidth at 4 V is at the magnetic gap distance, *s*, of 24.5 mm. At this gap of 24.5 mm, the responses of the BEH for different excitation amplitudes are studied and presented in Figure 17. It is clear that, with the increase of the excitation amplitude, the output voltage increases and the bandwidth is widened. Namely, the bandwidths at 4 V are 5 Hz, 8.5 Hz, and 9.5 Hz for excitation amplitudes of 2.5 m/s^2^, 3 m/s^2^ and 4 m/s^2^, respectively. The displacements of the second mass for a gap distance of 24.5 mm and 9 Hz for various excitation amplitudes are shown in Figure 18. Also, the phase portraits are plotted in Figure 19 to show that the snap through the potential wells is easier with increasing the excitation amplitude. The output voltages for the above condition are shown in Figure 20, from which it can be noted that the higher the excitation amplitude, the higher the output voltage. From the aforementioned simulation results, it can be concluded that, a gap distance of 24.5 mm can be considered for designing a BEH to achieve the widest bandwidth with the highest output voltage.

**Figure 21:**
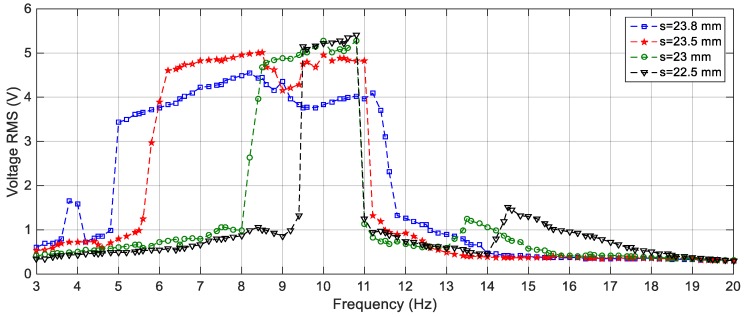
Experimental responses of the BEH for different gap distances, *s* = 23.8, 23.5, 23 and 22.5 mm at 3 m/s^2^.

**Figure 22:**
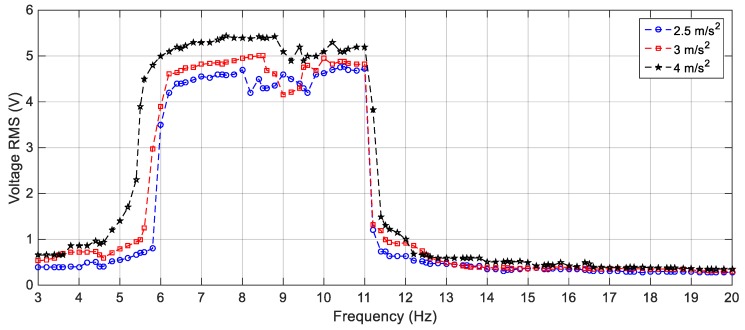
Experimental responses of the BEH for different excitation amplitudes (2.5 m/s^2^, 3 m/s^2^ and 4 m/s^2^), *s* = 23.5 mm.

On the other hand, the effect of varying the gap distance between the tip and the fixed magnets for the BEH are obtained experimentally and presented in Figure 21. It is considerably to note that, the experimental responses are shifted from the simulation ones; namely, the response of the BEH for *s* = 23.8 mm is the same as that of the simulated one for *s* = 24.2 mm. The experimental responses are shifted from the simulation ones. The reasons for that are the same as the ones of the linear response mentioned before. Also, the bi-stable effect started to appear at a smaller gap distance. This is due to simplification of considering the principle of dipole-dipole interaction to calculate the force between the tip and the fixed magnets. Additionally, the value of the magnetic flux density, *B_r_*, is assumed to be 1.2 T for simulation, while it ranges from 1.17 T to 1.22 T in the data sheet of the used magnets. Generally, by comparing Figure 16 with Figure 21, it is clear that the simulation and the experimental results have the same trend and the discrepancy is within a reasonable range. At a gap distance of 23.8 mm between the two repulsive magnets, a wide bandwidth of 6.6 Hz can be obtained for an output voltage of 3 V; while it is 2.6 Hz for 4 V. It can be noted that, the smaller the gap distance, the narrower the bandwidth and the higher the voltage. Specifically, the highest voltage recorded at *s* = 23.5 mm is 5 V at 8.5 Hz, while the bandwidth is 5 Hz for an output voltage of 4 V. Also, the effective bandwidth at 4 V is reduced to 2.4 Hz and 1.35 Hz for *s* = 23 mm and 22.5 mm, respectively. Additionally, the peaks at higher frequencies are recorded experimentally between 13 Hz and 15 Hz as clear in Figure 21. It can be concluded that, experimentally, a gap distance of 23.5 mm can be considered for designing a BEH to achieve the widest bandwidth at 4 V. In order to study the effect of varying the level of the excitation on the experimental response of the BEH, the gap distance is adjusted to 23.5 mm and the system is subjected to excitations of 2.5 m/s^2^, 3 m/s^2^ and 4 m/s^2^ and the recorded results are shown in Figure 22. As the excitation level increases, the mass acquires the energy to cross the potential barrier more easily and the piezoelectric patch generates more voltage.

#### 4.3.3. Quad-stable Energy Harvester

**Figure 23:**
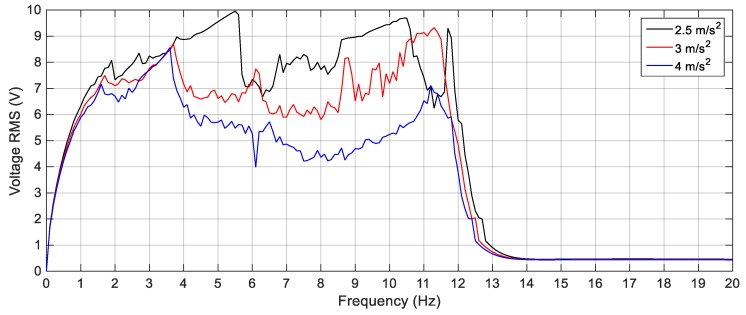
Simulation responses of the QEH for different excitation amplitudes (2.5 m/s^2^, 3 m/s^2^ and 4 m/s^2^), *s* = 22.8 mm and *w* = 10.84 mm.

**Figure 24:**
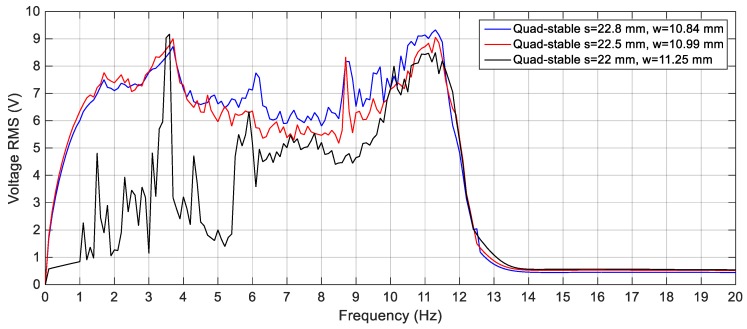
Simulation responses of the QEH for different magnetic gaps and excitation of 3 m/s^2^.

**Figure 25:**
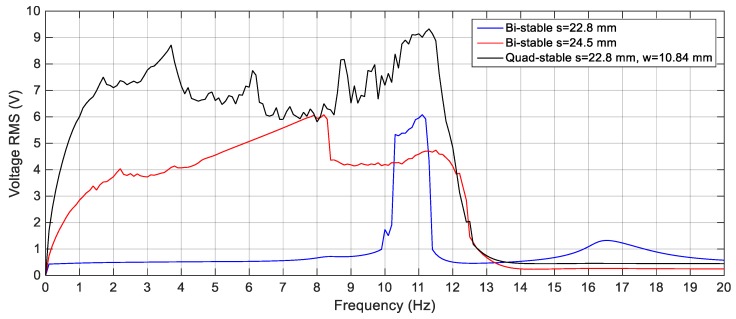
Simulation responses of the BEH for (*s* = 22.8 and 24.5 mm) and the QEH for (*s* = 22.8 mm and *w* = 10.84 mm) at 3 m/s^2^.

**Figure 26:**
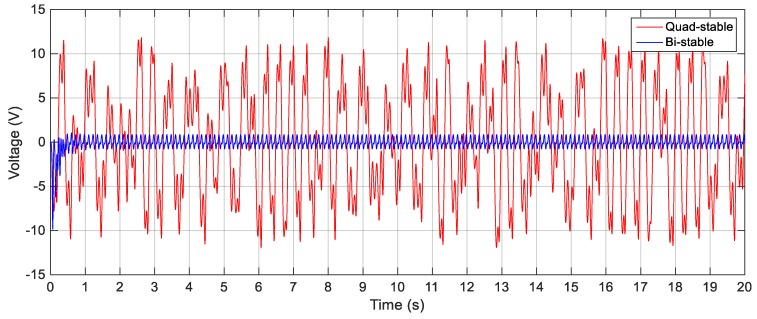
Simulated output voltages for the BEH and QEH at 3 m/s^2^ and 8 Hz for *s* = 22.8 mm and *w* = 10.84 mm.

**Figure 27:**
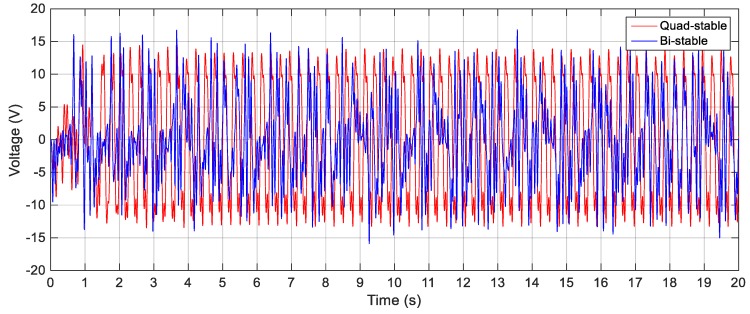
Simulated output voltages for the BEH and QEH at 3 m/s^2^ and 11 Hz for *s* = 22.8 mm and *w* = 10.84 mm.

To validate the superiority of the QEH over the BEH, three magnets are placed in front of the tip magnet to achieve the quad-stable configuration as clear in Figure 11b. The acceleration amplitudes of 2.5 m/s^2^, 3 m/s^2^ and 4 m/s^2^ are also used as a base excitation for the QEH. The simulation results of the RMS output voltages for *s* = 22.8 mm and *w* = 10.84 mm for different excitation amplitudes are shown in Figure 23, while the voltage responses of the QEH for other magnetic gaps are shown in Figure 24. It is clear that, the bandwidths of the QEHs for *s* = 22.8 mm and *w* = 10.84 mm and *s* = 22.5 mm and *w* = 10.99 mm are almost the same but, higher voltage generation can be obtained for *s* = 22.8 mm and *w* = 10.84 mm. To illustrate the improvement of the performance of the harvester due to the quad-stable configuration, the voltage response of the QEH at s = 22.8 mm, *w* = 10.84 mm and the BEH for the same gap distance of 22.8 mm between the tip and the fixed magnets, as well as for the gap distance of the optimal configuration of 24.5 mm and same excitation conditions of 3 m/s2 are compared in Figure 25. It is evident from Figure 25 that, the QEH can cover a wider bandwidth and generate higher voltages than the BEH. Also, the time responses of the output voltages for both the BEH at *s* = 22.8 mm and the QEH at 8 Hz and 11 Hz and excitation amplitude of 3 m/s^2^ are plotted in Figure 26 and Figure 27, respectively. At excitation frequency of 8 Hz, the second mass of the BEH is confined in one of the potential wells and the output voltage is very small, while that of the QEH snaps through among all the potential wells and the output voltage is very high. As the excitation frequency increases to 11 Hz, the second mass of the BEH can pass the potential barrier and the generated voltage increases so much, as clear in Figure 26. 

**Figure 28:**
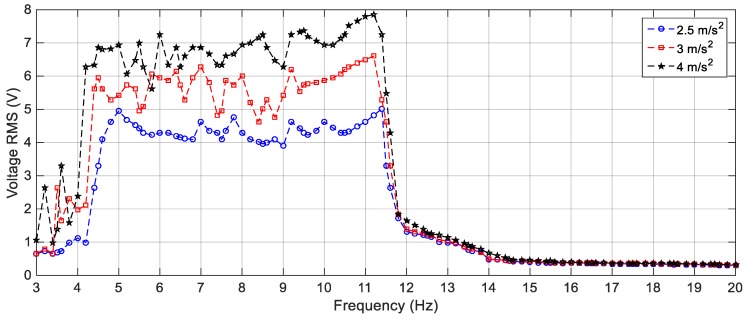
Experimental responses of the QEH for different excitation amplitudes (2.5 m/s^2^, 3 m/s^2^ and 4 m/s^2^), *s* = 22.8 mm and *w* = 9.9 mm.

**Figure 29:**
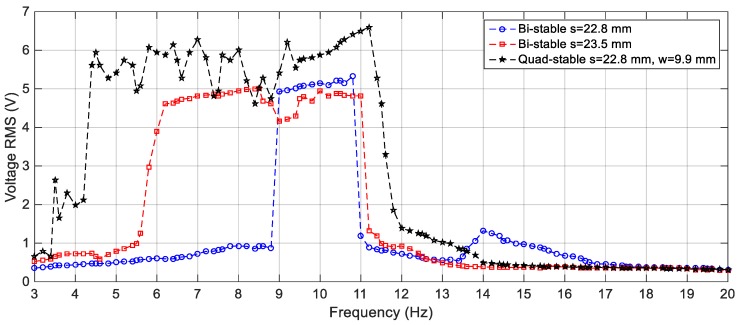
Experimental responses of the BEH for (*s* = 22.8 and 23.5 mm) and the QEH for (*s* = 22.8 mm and *w* = 9.9 mm) at 3 m/s^2^.

**Figure 30:**
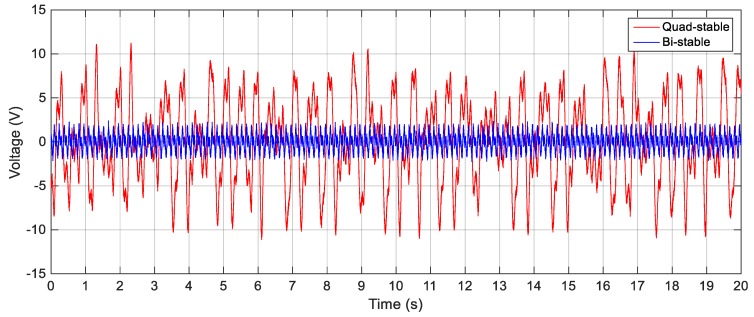
Experimental output voltages for the BEH and QEH at 3 m/s^2^ and 7.5 Hz for *s* = 22.8 mm and *w* = 9.9 mm.

**Figure 31:**
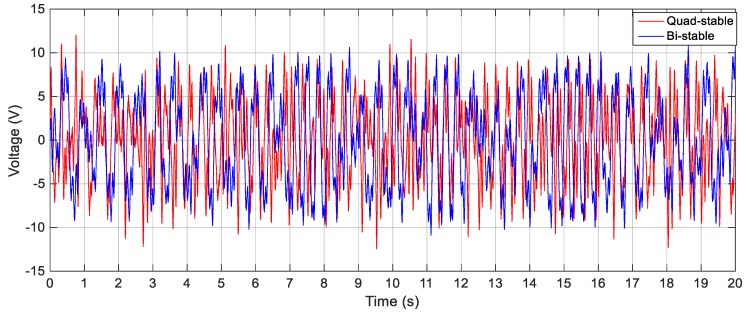
Experimental output voltages for the BEH and QEH at 3 m/s^2^ and 10 Hz for *s* = 22.8 mm and *w* = 9.9 mm.

In order to validate the simulation results, experiments are carried out for the same excitation levels; namely, 2.5 m/s^2^, 3 m/s^2^ and 4 m/s^2^. The gap distance between the tip and the fixed magnets is maintained at *s* = 22.8 mm and the quad-stable configuration is observed to have four equilibrium positions at *w* = 9.9 mm. Figure 28 shows the experimentally generated voltage of the QEH subjected to different excitation amplitudes of 2.5 m/s^2^, 3 m/s^2^ and 4 m/s^2^. Moreover, the experimental response of the QEH is compared with that of the BEH for the same gap distance, s, of 22.8 mm and s = 23.5 mm while maintaining the same excitation of 3 m/s2 as shown in Figure 29. It is clear that, the QEH is able to cover a wider bandwidth due to the capability of the second mass to travel among all the equilibrium positions more easily. For clarity, considering 4 V as the targeted output voltage, the QEH covers a bandwidth of 7.3 Hz, while the BEH bandwidth was 1.8 Hz and 5 Hz only for s = 22.8 mm and 23.5 mm, respectively. This proves that, the QEH can harvest vibration energy effectively more than the BEH. Furthermore, the time response of the scavenged voltage is compared for two excitation frequencies; namely, 7.5 Hz and 10 Hz as illustrated in Figure 30 and Figure 31. For lower frequencies at 7.5 Hz, the QEH shows chaotic motion among all the stable points which in turn leads to higher voltage generation, but the BEH at s = 22.8 mm is constrained in a potential well, and thus the output voltage was small. By increasing the frequency slightly to 10 Hz, the BEH performs interwell oscillation. Consequently, the generated voltage increases but, it is still lower than that of the QEH. 

In order to compare the performance of the proposed harvester with other harvesters reported in the literature, a figure of merit (FOM) should be identified. The FOM should take in to account the size of the harvester, the level of excitation, the effective bandwidth and the generated power. A FOM was proposed in [22] which has the form of:

(29) $$FOM=\frac{BW\times P_{Max}}{U^{2}}$$

where *BW* is the bandwidth normalized by the central frequency and can be calculated as *BW = (f_R_ − f_L_)/f_C_* in which *f_R_* and *f_L_* are the frequencies at half of the maximum power of the harvester and *f_C_* is the central frequency of this bandwidth, *P_Max_* is the maximum power of the harvester, *U* is the amplitude of the applied excitation. This FOM considers the maximum peak power which is not suitable for nonlinear harvesters with more than one peak and flat response. Moreover, the volume of the harvester was not considered, which makes this FOM inaccurate for comparing the performance of harvesters with different sizes. To overcome these shortcomings, this FOM should be modified to consider the volume of the harvester and a new figure of merit (FOM_new_) that expresses the density of the average power over a specific bandwidth versus the amplitude of the applied excitation and can be defined as:

(30) $$FOM_{new}=\frac{BW\times P_{avg}}{U^{2}\times V_{tot}}$$

where *P_avg_* is the average power of the harvester over the effective bandwidth and *V_tot_* is the total volume of the harvester. From (30) it is clear that, the higher the value of the average power and the wider the bandwidth, the higher the value of the FOM_new_ which means better performance of the harvester. Table 2 presents the FOM_new_ value for the proposed work and other previously reported harvesters. It is evident that the proposed QEH has the highest FOM_new_ among all the harvesters and the QEH achieves FOM_new_ that is 2.67 that of the one of the BEH.

## 5. Conclusions

A nonlinear BEH 2-DOF cut-out vibration energy harvester designed based on a systematic design methodology to scavenge vibration energy with a specific frequency range is introduced in this paper. This design methodology aims at widening the bandwidth of the harvester at a targeted output voltage. To achieve this target, the parameters of the harvester are chosen carefully to have specific natural frequencies. Also, the gap distances between the tip and the fixed magnets as well as the fixed magnets themselves are selected in the way that the potential energy curves have low potential barriers. Additionally, a QEH is developed to improve the performance of the BEH and covers a wider bandwidth. Numerical simulations are carried out to detect the optimal gap distances between the magnets that results in the widest bandwidth. A comparative study is presented to show the superiority of the QEH over the BEH in terms of the wider bandwidth and the higher RMS output voltages. Experiments are carried out at different harmonic excitations to validate the obtained simulation results. The results show that, the harvester designed based on the proposed methodology can cover a wide bandwidth. Moreover, the QEH can cover a wider bandwidth than that of the BEH and generate higher voltages for the same excitation. Additionally a proposed FOM is presented to compare the performance of the proposed configurations of the cut-out 2-DOF harvester with other harvesting devices. The QEH shows the highest value of the FOM among all harvesters and achieves 167% higher than that of the BEH configuration.

## Figures and Tables

**Table 1:** Dimensions and material properties of the harvester.

Parameter	Value	Parameter	Value
*L*_1_	93 mm	*C_p_*	115 nF
*L*_2_	50 mm	*λ*	12 × 10^−4^ N/V
*b*_1_	7 mm	*d*	10 mm
*b*_2_	20 mm	*µ*_0_	4π × 10^−7^ H m^−1^
*h*_1_	0.5 mm	*B_r_*	1.2 T
*h*_2_	0.2 mm	Magnet mass	7.5 g
*E*	210 GPa	Magnet size	20 × 10 × 5 mm^3^

**Table 2:** Performance comparison of this work with some of previously reported harvesters from the literature.

DOF	Structure	Source	Excitation (m/s^2^)	*f_R_ − f_L_* (Hz)	*f_C_* (Hz)	FOM_new_ (W·s^2^/m^4^)
SDOF	Cantilever beam	Ref. [41]	4.9	3	122	49.8 × 10^−3^
SDOF	Bi-resonant structure	Ref. [42]	10	15	20.5	0.1197 × 10^−3^
SDOF	Cantilever beam (Bi-stable)	Ref. [43]	10	2.75	12.625	63.62 × 10^−3^
SDOF	Cantilever beam (Quin-stable)	Ref. [44]	10	13	9.5	70.1 × 10^−3^
2-DOF	U-shaped structure (Nonlinear)	Ref. [45]	1	1.7	16.15	11.057 × 10^−3^
2-DOF	Magnetically coupled dual beam (Nonlinear)	Ref. [46]	3	2.2	14.9	129 × 10^−3^
2-DOF	Cut-out (Mono-stable)	Ref. [33]	2	5	17.3	19.12 × 10^−5^
2-DOF	Cut-out (Bi-stable)	This work	3	6.7	8.25	62 × 10^−3^
2-DOF	Cut-out (Quad-stable)	This work	3	7.3	7.95	165.9 × 10^−3^
